# Prevalence and Variations of the Median Artery: A Pilot Study in a Sample of Lithuanian Cadavers

**DOI:** 10.7759/cureus.57140

**Published:** 2024-03-28

**Authors:** Ignas Berškys, Andrej Suchomlinov

**Affiliations:** 1 Department of Anatomy, Histology, and Anthropology, Vilnius University Faculty of Medicine, Vilnius, LTU

**Keywords:** anatomical variations, cadaveric study, arteries of the upper limb, median artery, persistent median artery, palmar type, antebrachial type

## Abstract

Objective

This pilot project aimed to assess the prevalence and variations of the median artery (MA) on a small scale in preparation for a large-scale study investigating MA in Lithuanian cadavers.

Methods

Eight formalin-fixed adult female cadavers were used in this study. Dissection was performed to allow for the observation of MA presence, type, origin, termination, and relations with other structures. The gathered data was analyzed, and a literature search was performed to compare the findings.

Results

MA was found in 10 of the 16 upper limbs examined; therefore, the incidence of MA in the present study was 62.5%. Of the 10 MAs found, six (60%) were of the antebrachial type (a-MA), and four (40%) were palmar (p-MA). Thus, the prevalence of a-MA and p-MA in the upper limbs examined was 37.5% (N = 6/16) and 25% (N = 4/16), respectively. Among the six cadavers that were found to possess MA, it was identified bilaterally in four (66.7%) and unilaterally in two (33.3%). The associations between the antimere and the presence of MA or MA-type were not statistically significant. MA most commonly originated from the common interosseous artery (50%, N = 5/10), followed by the ulnar artery (UA) (40%, N = 4/10), and the anterior interosseous artery (10%, N = 1/10). Two (33.3%) of the six a-MAs terminated in the mid-forearm, while four (66.7%) a-MAs ended in the distal forearm. Meanwhile, three (75%) of the four p-MAs terminated by joining the UA, while one (25%) terminated as the first common palmar digital artery. In the forearm, nine (90%) of the 10 MAs traveled anteriorly to the anterior interosseous nerve (AIN), and only one (10%) traveled posteriorly to the AIN. Additionally, one (10%) of the 10 MAs was found to pierce the median nerve.

Conclusions

Our findings confirm the variability in MA characteristics reported by previous studies. The high incidence of MA discovered in our sample calls attention to the importance of being aware of MA in a clinical setting, as this would allow for a timely and accurate response to a potential pathology associated with this structure.

## Introduction

The median artery (MA) is generally only a transient vessel of the upper limb. It has been described as arising in early gestation and regressing as the radial and ulnar arteries develop [1]. However, MA might persist into adulthood. In this case, it presents as either of the two distinct types: antebrachial or palmar [2].

The antebrachial phenotype describes MAs that terminate in the forearm and are of minor size. Meanwhile, MAs of the palmar phenotype reach the hand and are quite sizeable [2]. Due to the high prevalence of the antebrachial type of MA (a-MA) in adults, it can be considered a regular trait [2,3]. The palmar type of MA (p-MA), on the other hand, is rarer and should be classified as an anatomical variant [2,3]. According to Natsis et al. [4], only p-MA should be known as a persistent median artery (PMA). MA has been found most commonly originating from the common interosseous artery (CIA), its terminal branch, the anterior interosseous artery (AIA), or the ulnar artery (UA) [5-7]. Antebrachial MAs have been observed positioned adjacent to the median nerve (MN) in the forearm and concluding either within the distal forearm (ending within the sheaths of the flexor tendons and persisting as only a slender branch in the connective tissue surrounding MN) or in the middle third of it (terminating inside the nerve sheath of MN) [2]. In the forearm, p-MA has been described as traveling towards the wrist in a connective tissue sheath shared with the median nerve (MN), surrounded by the forearm flexors both superficial and deep to it [8]. In the hand, p-MA can terminate as one or more common palmar digital arteries or unite with the superficial palmar arch [2].

The clinical importance of MA is highlighted by its relationship with various entrapment neuropathies of the upper limb [1,9,10]. MA, or exclusively the p-MA, has been assessed in a diverse group of cadaveric samples in, for instance, Brazil [5], Kenya [6], the USA [7], Japan [11], or South Africa [12,13]; however, never before in Lithuania.

The goal of this pilot research project was to evaluate the incidence and characteristics of MA in a sample of Lithuanian cadavers in preparation for a large-scale cadaveric study into the subject. It is also intended to compare the findings with those published by other authors and describe the clinical significance of MA.

## Materials and methods

This study was completed at the Department of Anatomy, Histology, and Anthropology, Vilnius University Faculty of Medicine, Lithuania. Eight formalin-fixed adult female cadavers were used for dissection. Only cadavers without any visible trauma or pathology of the upper limbs bilaterally were included. All of the donors have provided written informed consent (using signing special notaries' approved forms) to allow for the conduct of scientific research with their bodies after death. Additionally, permission to conduct this study (No. 57) was received from the Lithuanian Bioethics Committee. The study was carried out in accordance with the Helsinki Declaration.

**Figure 1:**
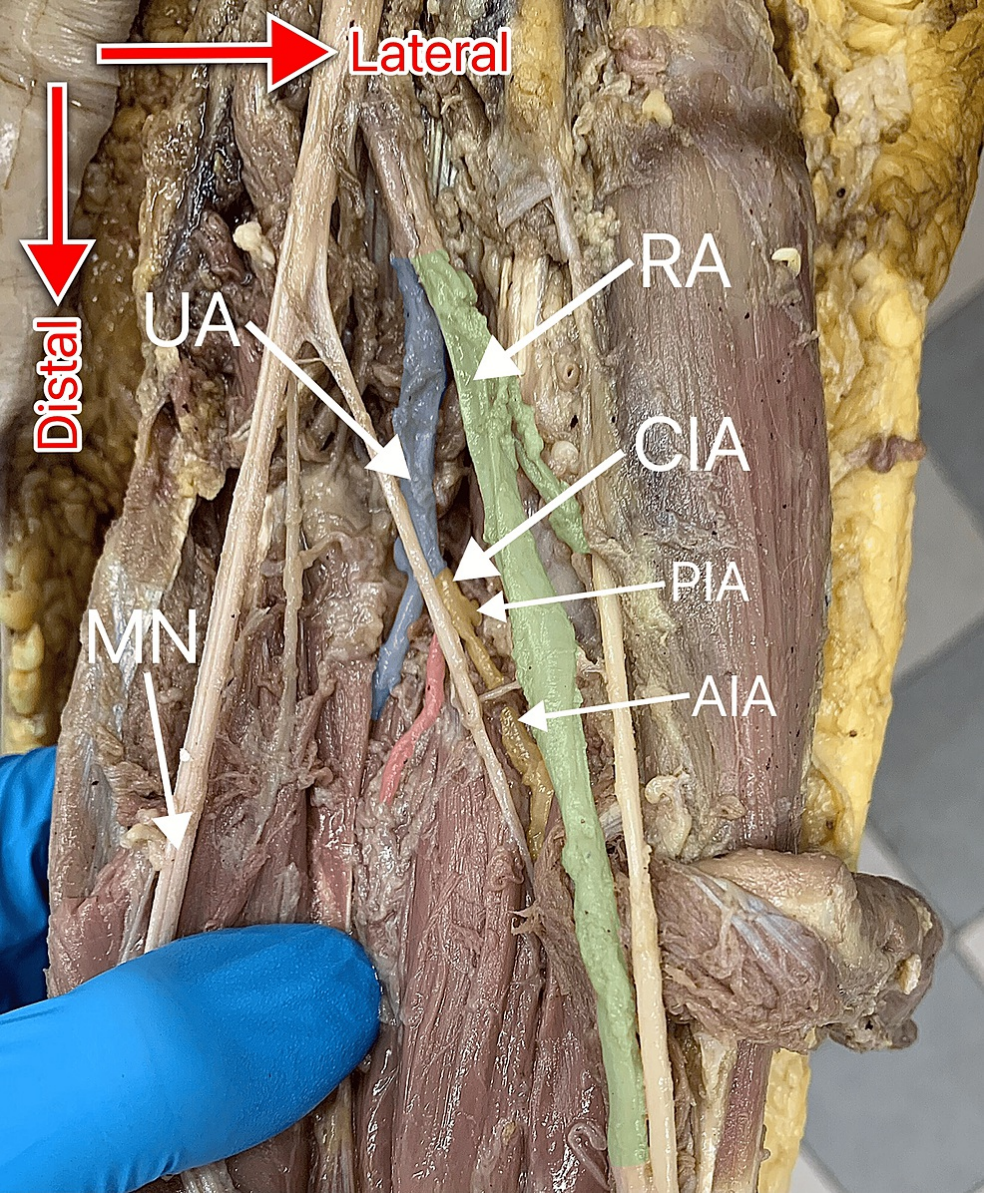
Arteries in the proximal forearm as seen on dissection. The median artery is absent. UA: ulnar artery (colored blue); RA: radial artery (colored green); CIA: common interosseous artery (colored yellow); AIA: anterior interosseous artery (colored yellow); PIA: posterior interosseous artery (colored yellow); MN: median nerve; colored red: a muscular branch stemming from CIA

The dissection protocol employed was as follows: firstly, a transverse incision was made at the level of the mid-upper arm, which was subsequently connected with a longitudinal incision running down the center of the anterior surface of the forearm. Both the skin and the subcutaneous tissue were cut and reflected. The antebrachial fascia was removed as needed, followed by separation and retraction of the forearm muscles. The pronator teres muscle was transected, and the flexor digitorum superficialis muscle was cut longitudinally, thus allowing for the visualization of MN along its course and of the arteries in the proximal forearm. Namely, the radial artery (RA), the ulnar artery, the common interosseous artery, and its terminal branches, the anterior and posterior interosseous arteries, were identified. Any additional vessels originating in this locale were also noted (Figure 1).

If a vessel accompanying MN was observed, it was treated as MA. In such instances, the previously mentioned longitudinal incision was extended distally to track the vessel. The origin of MA was investigated as well. In the case of MA reaching the wrist, the transverse carpal ligament was divided as described in Grant’s Dissector [14], so as not to injure the underlying structures. Afterward, the soft tissues of the hand, including the palmar aponeurosis, were dissected to expose the superficial arterial vasculature of the palm and to witness the fate of MA. Findings about the incidence of MA, its type, origin, termination, and relation to the anterior interosseous nerve (AIN), as well as any instances of MA piercing MN, were documented and photographed.

Data gathered was analyzed using Microsoft Excel 2021 for Mac (Microsoft® Corp., Redmond, WA) and RStudio Desktop version 2023.12.1+402 (Posit Software, PBC, Boston, MA) with “Rcmdr” package version 2.9-1 [15] installed in R version 4.3.2 (R Foundation for Statistical Computing, Vienna, Austria). Descriptive statistical analysis and nonparametric statistical testing were carried out. Namely, Fisher’s exact test was used to check for an association between these categorical variables: the antimere examined, the presence of MA, the phenotype of MA, and the MA origination variant. Results were treated as statistically significant if the p-value was less than 0.05. Figures presented in this article have been edited by digitally coloring the structures of interest using the Markup tool on iPadOS Version 17.3 (Apple Inc., Cupertino, CA). This was done to highlight these structures and emphasize their relations. A literature search and analysis were performed to contextualize the findings and compare them to earlier studies.

## Results

**Figure 2:**
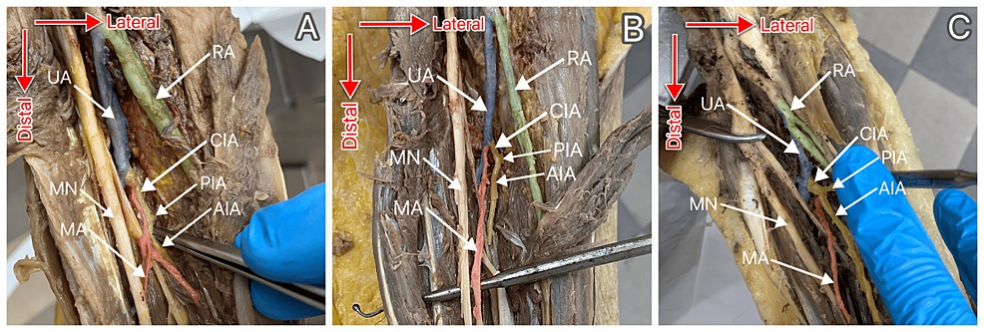
Different origination variants of the median artery were observed. A: MA originating from the CIA; B: MA originating from the UA; C: MA originating from the AIA UA: ulnar artery (colored blue); RA: radial artery (colored green); CIA: common interosseous artery (colored yellow); AIA: anterior interosseous artery (colored yellow); PIA: posterior interosseous artery (colored yellow); MA: median artery (colored red); MN: median nerve

16 upper limbs of eight cadavers were examined for the presence of MA. All of the donors happened to be of the female sex, with an average age of 75.5 years (age range: 37-92 years). 10 out of the 16 cadaveric upper limbs examined were found to possess MA, giving an incidence of this artery in the present study of 62.5%. MA was present in six (75%) of the eight left upper limbs and four (50%) of the eight right upper limbs. The association between the antimere and the presence of MA was not statistically significant (p = 0.608). Six (60%) of the 10 MAs observed were of the antebrachial type, and four (40%) of the 10 were classified as palmar, giving the prevalence of a-MA and p-MA in the upper limbs examined of 37.5% (N = 6/16) and 25% (N = 4/16), respectively. MA was identified in six (75%) of the eight cadavers and in four (66.7%) of those bilaterally. In four cases where MAs were present bilaterally, twice (50%) were they both antebrachial, once (25%) both were palmar and in one case (25%), MA on the left side was palmar, while on the right side was antebrachial. On the left side, MAs were of the antebrachial type in four (66.7%) of the six cases and of the palmar type in two (33.3%). On the right side, the same number of MA cases (two of each) belonged to the antebrachial and palmar types. The association between the antimere and the type of MA present was not statistically significant (p = 0.999). It was also noted that MA most commonly originated from the CIA. This was recorded in five (50%) of the 10 upper limbs in which MA was found. MA originated from UA in four (40%) and from AIA in one (10%) of the 10 upper limbs in which MA was found. The different MA origination variants observed are represented in Figure 2.

Antebrachial MAs most often stemmed from CIA. Palmar MAs stemmed from CIA and UA equally as often, without ever originating from AIA. A statistically significant association between MA type and MA origination variant could not be established (p = 0.999). A detailed breakdown of the distribution of MA origination variants among the cases belonging to each of the phenotypes is displayed in Table 1.

**Table 1:** Distribution of MA origination variants among the cases belonging to the antebrachial and the palmar phenotypes. Data represented as frequency count (n) and percentage (%). P-value < 0.05 was considered significant. MA: median artery; CIA: common interosseous artery; UA: ulnar artery; AIA: anterior interosseous artery

MA Phenotype	MA Origination Variants						P-value
	Originating from CIA		Originating from UA		Originating from AIA		0.999
	Frequency (n)	Percentage (%)	Frequency (n)	Percentage (%)	Frequency (n)	Percentage (%)	
Antebrachial	3	50	2	33.3	1	16.7	
Palmar	2	50	2	50	0	0	

MAs in the left upper extremity most often arose from CIA, while in the right upper extremity, the most common vessel of origin for MA was UA. Again, there was no association between the two variables (p = 0.191). Data on the distribution of MA origination variants in cases of MA in each of the antimeres is provided in Table 2.

**Table 2:** Distribution of MA origination variants in cases of MA in the left and the right upper limbs. Data represented as frequency count (n) and percentage (%). P-value < 0.05 was considered significant. MA: median artery; CIA: common interosseous artery; UA: ulnar artery; AIA: anterior interosseous artery

Antimere	MA Origination Variants						P-value
	Originating from CIA		Originating from UA		Originating from AIA		0.191
	Frequency (n)	Percentage (%)	Frequency (n)	Percentage (%)	Frequency (n)	Percentage (%)	
Left Upper Limb	4	66.7	1	16.7	1	16.7	
Right Upper Limb	1	25	3	75	0	0	

**Figure 3:**
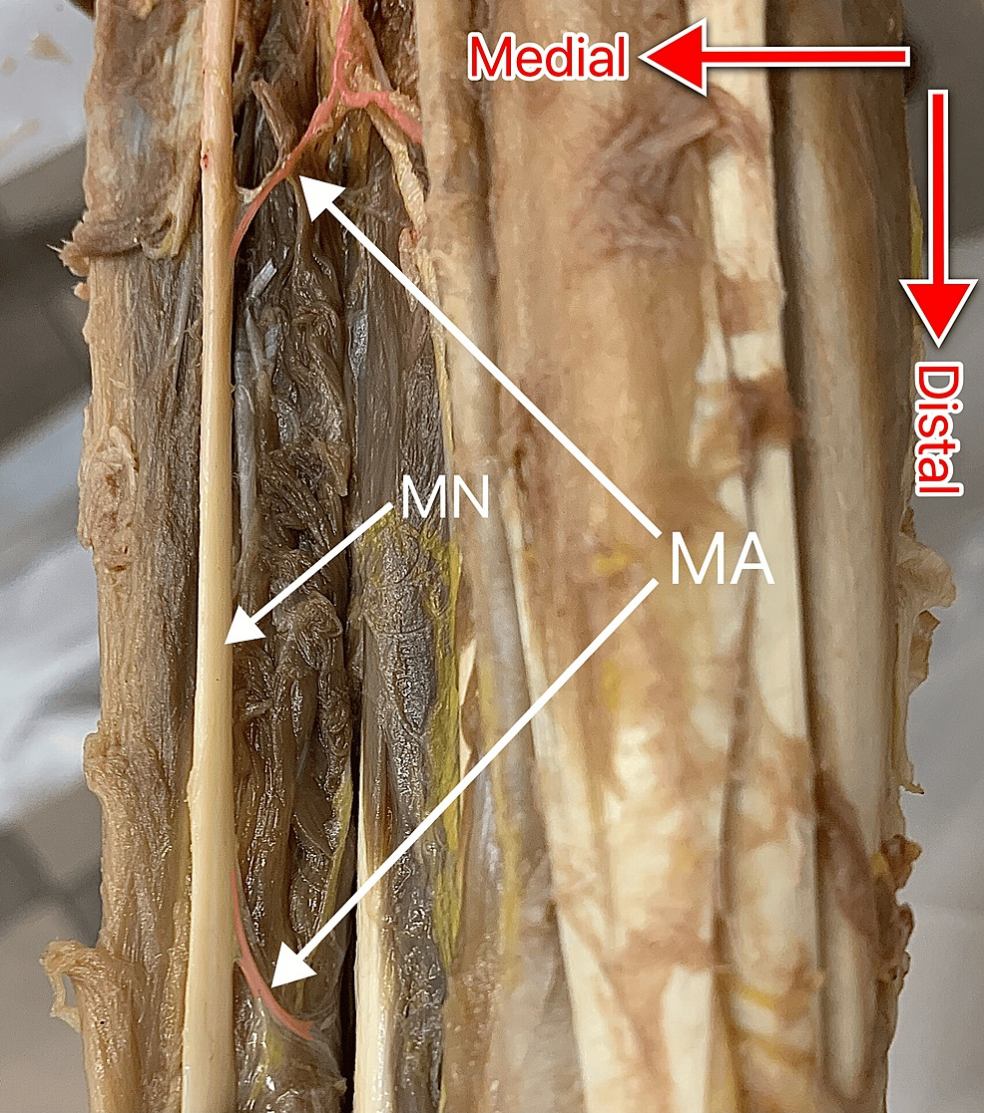
Median artery terminating between the fibers of a muscle in the mid-forearm. MA: median artery (colored red); MN: median nerve

Two (33.3%) of the six a-MAs diverged from MN and terminated between the fibers of the adjacent muscle in the mid-forearm (Figure 3).

**Figure 4:**
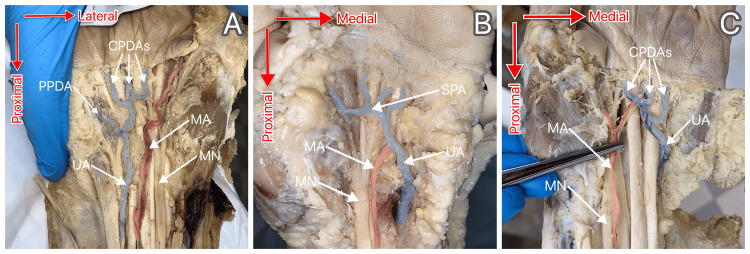
Different termination variants of palmar MA were observed. A: MA terminating as the first common palmar digital artery; B: MA flowing into the ulnar artery; C: MA anastomosing with the ulnar artery via a branch and continuing towards the first web space UA: ulnar artery (colored blue); CPDAs: common palmar digital arteries (colored blue); PPDA: proper palmar digital artery to the fifth digit (colored blue); SPA: superficial palmar arch (colored blue); MA: median artery (colored red); MN: median nerve

The remaining four (66.7%) of the six a-MAs terminated in the distal forearm. Once (25%) they did so in the tissue surrounding MN, once (25%) in the tissue surrounding the flexor tendons, and twice (50%) in the tissue surrounding MN while giving off a branch ending in the tissue surrounding the flexor tendons. Meanwhile, three (75%) of the four p-MAs joined UA, while one (25%) terminated as the first common palmar digital artery (CPDA) without joining UA. In the three cases of MA joining UA, it joined via a branch twice (66.7%) and by merely flowing into it once (33.3%). Termination variants of palmar MA observed are represented in Figure 4.

In the forearm, MA traveled anteriorly to AIN in nine of the 10 upper limbs (90%) and posteriorly to it in one (10%). One (10%) of the 10 MAs discovered pierced MN in the forearm. This MA happened to be of the palmar type. Several instances of MA-giving branches that supplied the surrounding muscles were noticed. However, due to the minuteness of those branches, their incidence was not recorded since this could not be done reliably in all cases.

## Discussion

To preface the following comparisons between the findings of the current study and those performed previously, it is important to highlight that the varying definitions of MA and PMA used made these more challenging. For example, in one study, Henneberg and George [13] treated MA as being present only if its minimal diameter was greater than 1 mm, it exceeded 2 mm in diameter at the point of origin, and it irrigated the hand. According to them, these criteria distinguish MA from *arteria comitans nervi mediani* [13]. Rodríguez-Niedenführ et al. [2], on the other hand, consider the terms *arteria comitans nervi mediani* and MA to be synonymous. In their study, the authors did not employ the diameter criteria for MA and recorded both the large arteries that reached the hand and the small ones that did not [2]. Also, some authors treated only the palmar type of MA as PMA [4,11], while others seemed to be referring to both MA phenotypes when using the term [7,16]. This made it difficult to discern whether the authors of a particular study investigated both MA phenotypes or only p-MA. The terminology around MA and PMA needs more clarity.

To our knowledge, this study marks the first time MA has been studied in a sample of Lithuanian cadavers. Despite the small sample size, we were able to appreciate both types of MA, different variants of MA origin, and varying termination patterns of both the antebrachial and the palmar phenotypes. In this study, we found MA with an incidence of 62.5% (in 10 of the 16 upper limbs), which was comparable to that reported by Cheruiyot et al. [6] in Kenya, who identified MA in 37 (59.7%) of the 62 limbs. We identified MA more often than Saenz et al. [7] in the USA, who found MA in 25 (20.8%) of the 120 upper limbs, or Lucas et al. [17] in Australia, who found MA in 26 (33.3%) of the 78 upper limbs, yet less frequently than Aragão et al. [5] in Brazil, who found MA in 26 (81.3%) of the 32 upper limbs. A study by D’Costa et al. [18], which investigated the occurrence of p-MA exclusively, has suggested that the observed differences between various populations regarding the prevalence of the artery might be caused by allelic variations in its regulatory genes. Lucas et al. [17] have proposed that the differences in the incidence of MA seen between samples of similar age from various locales might be explained by the varying methodologies used. We believe that a combination of these factors could explain the discordance in MA prevalence between the previously referenced studies and the current one. Moreover, Aragão et al. [5] investigated the prevalence of MA in fetuses (average gestational age of 30 weeks), not adults. The authors raised the theory that the regression of MA might continue into the latter stages of gestation [5]. This could explain the higher incidence of MA in their study as compared to that found in ours since we investigated MA in adult cadavers.

We found MA in the left upper limb more often than in the right upper limb. However, this was not found to be statistically significant. The lack of statistical significance in the difference between the prevalence of MA in the left and right upper limbs was also reported by Cheruiyot et al. [6]. We found MA bilaterally more frequently than unilaterally (in four (66.7%) and two (33.3%) of the six MA-positive cadavers, respectively), while Saenz et al. [7] found MA presenting bilaterally and unilaterally at a similar rate (in eight (47.1%) and nine (52.9%) of the 17 MA-positive cadavers, respectively). In our study, CIA was the most common source of MA, a finding shared by the studies of Aragão et al. [5] and Cheruiyot et al. [6]. Meanwhile, UA was the most common vessel of origin for MA in the study by Saenz et al. [7]. We were unable to identify a statistically significant association between the antimere or MA phenotype and the MA origination variant. A lack of statistically significant association between MA phenotype and MA origination variant has also been noted by Saenz et al. [7].

We observed 90% (N = 9/10) of MAs passing anteriorly to AIN and only 10% (N = 1/10) passing posteriorly to it. Similar findings were made by Matsunaga et al. [19] (80.6% (N = 79/98) and 10.2% (N = 10/98), respectively). Matsunaga et al. [19] also reported instances of MA traveling between the branches of AIN and a single instance (1%, N = 1/98) of MA piercing AIN; we did not come across such cases. We observed only one (10%) of the 10 MAs piercing MN. Meanwhile, Saenz et al. [7] found this to be true in 15 (60%) of the 25 MA cases. They also discovered that MN was more often pierced by p-MA than a-MA [7]. The singular MA that pierced MN in our study was also of the palmar type. Our observation that MA occasionally gives branches to supply the surrounding muscles while traveling down the forearm is echoed by Rodríguez-Niedenführ et al. [2].

In our study, a-MA was found in six (37.5%) of the 16 upper limbs dissected. This is substantially lower than the prevalence of 98% (N = 98/100) reported by Matsunaga et al. [19] or the 53.2% (N = 84/158) found by Rodríguez-Niedenführ et al. [2]. However, our findings are almost identical to those of a meta-analysis performed by Solewski et al. [16]. It reported a pooled prevalence of a-MA of 38.5% using data from six cadaveric studies, which included a total of 409 upper limbs [16]. In our study, a-MA most often originated from CIA, while in the studies by Rodríguez-Niedenführ et al. [2] and Saenz et al. [7], a-MA most commonly stemmed from AIA. It is worth noting that the only case of MA originating from AIA in this study was also of a-MA. A study by Khan and Shrestha [20] (which found a-MA in six (12%) of the 50 upper limbs) noted the posterior interosseous artery as the most common vessel of origin for a-MA. We did not come across such a variant during dissection. Our findings on the termination of a-MA add to those of Rodríguez-Niedenführ et al. [2]. As in their study, antebrachial MAs in ours terminated either in the mid-forearm (MF) (33.3%, N = 2/6) or the distal forearm (DF) (66.7%, N = 4/6), though at different rates (compare 73.8% (N = 62/84) of a-MAs ending in MF and 26.2% (N = 22/84) ending in DF as reported by Rodríguez-Niedenführ et al. [2]). Another point of distinction is that a-MAs that ended mid-forearm in our study did so by terminating between the fibers of the adjacent muscle, while in the study by Rodríguez-Niedenführ et al. [2], they did so by ending in the sheath of MN.

Incidence of p-MA in the present study (found in four (25%) of the 16 upper limbs examined) was higher than that observed by Natsis et al. [4] (2.8%, N = 2/72), Eid et al. [11] (4%, N = 2/50), Patnaik and Paul [8] (6%, N = 6/100), Singla et al. [21] (6.7%, N = 4/60), Claassen et al. [22] (7.4%, N = 4/54), Saenz et al. [7] (10%, N = 12/120), Rodríguez-Niedenführ et al. [2] (12.1% N = 29/240), Nayak et al. [23] (15.5% N = 13/84), D’Costa et al. [18] (15.8% N = 6/38), or Cheruiyot et al. [6] (19.4% N = 12/62). Khan and Shrestha [20] and Matsunaga et al. [19] did not document any MAs of the palmar type, despite dissecting 50 and 100 upper limbs, respectively. The previously mentioned meta-analysis by Solewski et al. [16] calculated the pooled prevalence of p-MA of 8.6% (N = 310/3590) using data from 39 studies on adult cadavers. The only studies identified that dealt with adult cadavers and recorded a similar p-MA prevalence were those of Olave et al. [24] (22.5%, N = 23/102) and Henneberg and George [12,13] (27.1%, N = 26/96, and 27.4%, N = 17/62, respectively). Although these studies did not use the term palmar median artery, they documented MAs that entered the hand; therefore, those vessels could be classified as such. A study by Lucas et al. [17] discovered that the prevalence of MA in people born in the 1990s was about three times higher than that observed in people born in the mid- or late-19th century. The authors theorized that the observed trend could be explained by the shift in health insults, as experienced by both fetuses and their mothers, that might inhibit the involution of MA or, alternatively, by the process of evolution. [17]. We wonder if the same trend exists for p-MA specifically and could explain, at least in part, the relatively high incidence of p-MA in this most recent study as compared to most of the previously performed studies. No single origination variant of p-MA dominated our study (50% (N = 2/4) stemmed from CIA and 50% (N = 2/4) from UA). Henneberg and George [13] found MAs (which we would consider to belong to the palmar type) most commonly stemming from AIA. Rodríguez-Niedenführ et al. [2] observed p-MAs most often originating from the caudal angle between UA and CIA. Saenz et al. [7] noted that p-MA most frequently originated directly from UA. Meanwhile, CIA was the most common vessel of origination for p-MA in the study by Nayak et al. [23]. From this, it is clear that the origin of p-MA can be quite varied. It is worth mentioning that we have not observed p-MA stemming from AIA. Also, although we have discovered MAs originating from the caudal angle between UA and CIA, we have classified these as stemming from UA. Only one (25%) of the four p-MAs observed did not anastomose with UA and continued as the first common palmar digital artery (CPDA) in the present study. The remaining three (75%) of the four p-MAs anastomosed with UA by either directly flowing into it or via a branch. Both p-MAs that anastomosed with UA and those that did not and continued as one or more CPDAs have been documented in the previous studies [2,6,13,22,23,24].

Clinical significance

A majority of PMAs do not cause any symptoms [25]. A case of anterior interosseous nerve syndrome associated with MA has been described by Proudman and Menz [9]. In this case, the piercing of AIN by MA was evident intraoperatively [9]. Meanwhile, a case of pronator teres syndrome caused by PMA has been reported by Jones and Ming [1]. Here, carpal tunnel release surgery had been previously performed on both hands and PMAs bilaterally have been noted. Subsequent to the return of symptoms on the right side, exploratory surgery was performed, and a median artery penetrating MN in the forearm and multiple *rr. musculares *that pressed on the nerve were observed [1]. Boles et al. [10] have also described a patient with carpal tunnel syndrome (CTS) who was found to have a large PMA intraoperatively. This was attributed to the cause of the patient’s ailment [10]. Aneurysm [26] or thrombosis [27] of PMA have also been previously described as leading to CTS. Yet curiously, Solewski et al. [16] theorized that possessing a PMA might ensure a superior blood supply to MN, thus safeguarding against CTS development. Their meta-analysis found p-MA to be more than three times as prevalent among the hands of adult cadavers than among patients receiving CTS surgery [16]. As carpal tunnel release by itself is usually sufficient for symptomatic relief, Bilgin et al. [28] recommended abstaining from transposing or resecting PMA during surgery for CTS if no further abnormalities are present. According to Zeiss and Guilliam-Haidet [29], determining the contributions to the arterial supply of the hand by the median, ulnar, and radial arteries should precede PMA resection if one is intended to be done, as this could risk digital ischemia. Thrombosis of PMA leading to ischemia of the digits has also been documented [30]. Moreover, Natsis et al. [4] have suggested that PMA might be utilized as a vascular graft, while Davidson and Pichora [25] reported on the successful use of a forearm fasciocutaneous free flap supplied by PMA.

Limitations of the study

This study, being a pilot project and dealing with cadaveric material, which is so often of limited availability, suffers from a small sample size. The results of this study cannot be treated as representative of Lithuania’s population more broadly or even Lithuania’s cadaveric population more specifically. Also, all of the donors included in the present study happened to be female. Therefore, no comparison between the presence and characteristics of MA in males and females could be made. Moreover, since this study dealt with small, sometimes translucent vessels, the risk of inadvertent damage was high. This could have led to an underestimation of the incidence of MA and an incorrect identification of its characteristics. We are exploring the use of additional techniques, such as latex injection, as was employed by Aragão et al. [5], to help with the visualization of these intricate structures. Due to the formalin-caused overhardening of tissue, the dissection adequate enough for the observation of the relationship between MA and RA could not be performed. This prohibited us from classifying the superficial palmar arches (SPAs) observed. Although rare, radial-median-ulnar and median-radial SPAs have been reported by Nayak et al. [23], with an incidence of 3.6% (N = 3/84) and 1.2% (N = 1/84), respectively. We are investigating the use of other preservation methods, such as ethanol-glycerin fixation combined with thymol conservation [31], for our prospective large-scale cadaveric study on MA.

## Conclusions

Our findings confirm the variability of MA origination and termination, as described by earlier studies. The discovery of MA in the majority of upper limbs and the finding of p-MA in the quarter of the hands examined highlight the need for knowledge pertaining to MA in clinical practice. Awareness of MA and its variations is crucial to ensure prompt diagnosis and appropriate treatment of potential pathologies of this structure. Moreover, when evaluating patients with suspected entrapment neuropathies of the median and the anterior interosseous nerves, their potential relationship with the median artery must be kept in mind.
